# The "Cardiac Sign" of Malignant Disease

**Published:** 1914-04-25

**Authors:** 


### MODERN METHODS SERIES.

### The "Cardiac Sign" of Malignant Disease.

With unwearied energy Dr. W. Gordon continues to proclaim his faith in a particular physical sign which he believes to be strongly indicative of malignant disease. Thus in this present year he has already contributed to the *Lancet*,* the *Practitioner*† and the *Bristol Medico - Chirurgical Journal*‡ on this subject. Dr. Gordon is honorary physician to the Royal Devon and Exeter Hospital, and his "sign" consists in a marked diminution of the area of cardiac dulness in the recumbent posture as determined by digital percussion. When this lessened cardiac dulness is found, without other obvious explanation, he holds that in the large majority of cases the patient will be found to be suffering from cancer in some region of the body, and the sign is of especial value if the stomach be the organ in which a tumour is suspected. The absence of the sign, too, is contra-indicative.

There are not wanting sceptics who deny the validity of Dr. Gordon's contentions about his "sign." Scientific scepticism is a good thing, and so is enthusiasm in pursuit of a new discovery. Very often the truth is ultimately found to lie somewhere midway between the two extremes, and in any case it is unwise to pin one's faith to the observations of a single clinician on any medical question. Let us briefly, first, therefore, ascertain exactly what Dr. Gordon's claims are, and then endeavour to reason out any possible sources of fallacy underlying them.

In the first place it is important to notice that the patient must be recumbent before the "cardiac sign" is sought for. In the normal adult the cardiac dulness in this posture begins above at about the third left costal cartilage, is bounded on the right by the midline of the sternum, and measures about three to three and a half inches in width at the level of the fifth costal cartilage. In carcinoma cases, according to Dr. Gordon, it will often be found that the dulness in recumbency begins above at about the fourth or the fifth costal cartilage, has its right edge half an inch or an inch to the left of the midsternal line, and measures only two inches, or even much less, in width at the level of the fifth costal cartilage; sometimes there is no cardiac dulness at all.

Certain obvious limitations of this sign are admitted by Dr. Gordon. Thus ordinary emphysema, as is well known, has a great tendency to obliterate the cardiac dulness, wholly or in part; and in emphysematous patients, accordingly, the sign is of no value. Inasmuch as the age distribution of cancer is not very different from that of emphysema, this discounts the helpfulness of the "cardiac sign" in a very considerable proportion of cases. Then, again, some conditions lead to marked increase in the cardiac dulness: such are chronic Bright's disease, chronic valvular disease of the heart, pericarditis, old fibrous phthisis, and old pleurisy. If any of these conditions are present the absence of the sign is correspondingly deprived of any significance. Also when the heart for any reason is displaced upwards—as for instance by ascites—the absence of the sign is unreliable.

It is unnecessary to discuss the theoretical basis of this alleged diminution of cardiac dulness in cases of malignant disease. Dr. Gordon has three hypothetical explanations, none of which is very convincing. It is, however, legitimate to point out that cardiac dulness is a matter very largely of opinion, and that clinicians of the highest eminence often differ very markedly as to its exact boundaries in individual cases. The percussion of cardiac dulness, in fact, is a much more difficult and "tricky" business than the percussion of pulmonary dulness (or resonance). The personal element, that is, enters very largely into all determinations of the cardiac percussion note. Next, it is noteworthy that the extent of cardiac dulness varies a good deal among healthy individuals; there are a good percentage who do not conform to the normal as laid down by Dr. Gordon. Here, then, are two very serious elements of possible error.

Emphysema, again, is a very common condition among elderly people, and it is not rare, indeed, at any age. In advanced emphysema the diagnosis is easy, no doubt, and the exclusion of such cases presents no difficulty. But there are plenty of patients with early emphysema in whom there is little sign of it, other than diminution of cardiac dulness, and this introduces at once a source of very perceptible confusion.

In compiling his statistics Dr. Gordon claims to have excluded all cases in which any of the admitted sources of fallacy might vitiate the result. His latest returns show that of 111 cases of cancer, 97 exhibited the cardiac sign (87 per cent.), whereas among 107 non-cancerous cases 89 failed to exhibit it (84 per cent.). It will thus be seen that even Dr. Gordon is misled by his own sign in something like one case out of seven; and it is not unlikely that others less familiar with it might find it even less reliable. In sarcoma, it may be added, Dr. Gordon finds it of much less utility than in carcinoma. Nor does it appear, according to his observations, at an early stage of malignant disease.

It will be gathered that, even if other workers confirm Dr. Gordon's results, this sign is not of the same order of trustworthiness as the Widal reaction in typhoid fever, the loss of liver dulness in perforated gastric ulcer, the Babinski reflex in meningitis, and similar more or less pathognomonic signs. In fact, its usefulness appears to be very strictly limited indeed. If the experience of other observers is less favourable than that of the inventor, and if the hypothetical objections already discussed are well founded, the "cardiac sign" may turn out to be so ambiguous in its bearings upon diagnosis as to be of absolutely no value whatever. In any case, there is plenty of room for independent research into the matter whereby sufficient statistics can easily be accumulated for definite conclusions to be drawn.

* January 17, 1914, p. 161. † April 1914, p. 586. ‡ March 1914, p. 19.

